# Macrophages Recognize Size and Shape of Their Targets

**DOI:** 10.1371/journal.pone.0010051

**Published:** 2010-04-06

**Authors:** Nishit Doshi, Samir Mitragotri

**Affiliations:** Department of Chemical Engineering, University of California Santa Barbara, Santa Barbara, California, United States of America; Charité-Universitätsmedizin Berlin, Germany

## Abstract

Recognition by macrophages is a key process in generating immune response against invading pathogens. Previous studies have focused on recognition of pathogens through surface receptors present on the macrophage's surface. Here, using polymeric particles of different geometries that represent the size and shape range of a variety of bacteria, the importance of target geometry in recognition was investigated. The studies reported here reveal that attachment of particles of different geometries to macrophages exhibits a strong dependence on size and shape. For all sizes and shapes studied, particles possessing the longest dimension in the range of 2–3 µm exhibited highest attachment. This also happens to be the size range of most commonly found bacteria in nature. The surface features of macrophages, in particular the membrane ruffles, might play an important role in this geometry-based target recognition by macrophages. These findings have significant implications in understanding the pathogenicity of bacteria and in designing drug delivery carriers.

## Introduction

**Figure 1:**
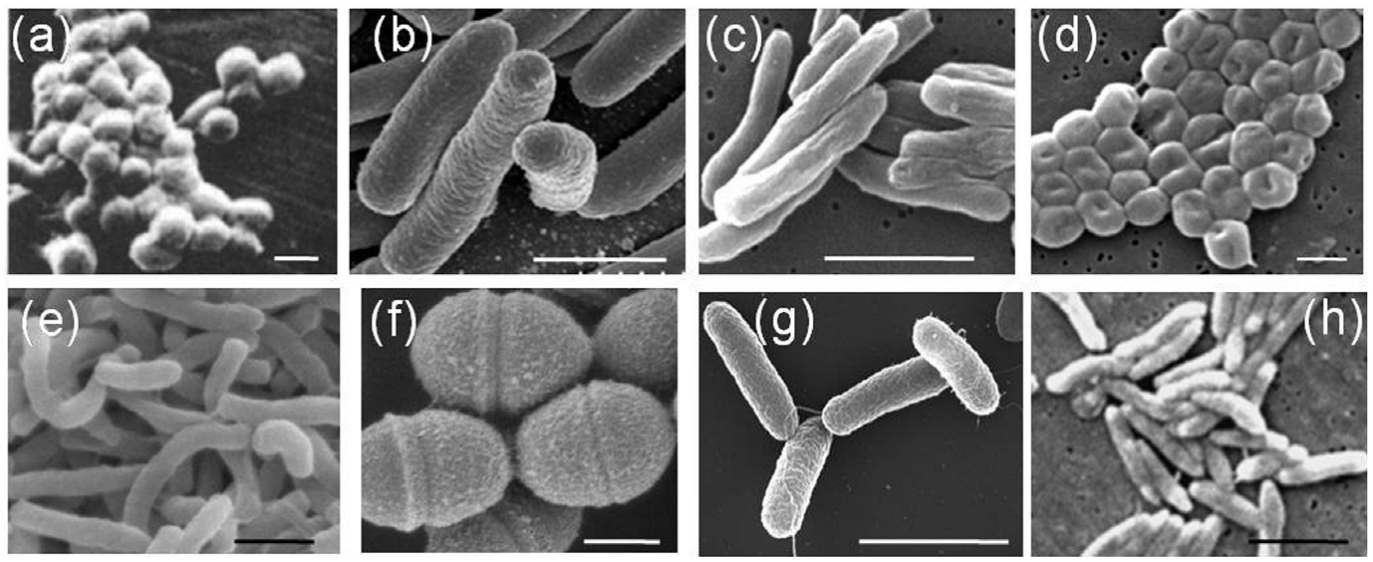
Bacteria exhibiting a wide variety of sizes and shapes. (a) Mycoplasma pneumonia (spherical)[28] (b) Escherichia coli (Rod shaped)[29] (c) Mycobacterium tuberculosis (Rod shaped)[30] (d) Acinetobacter Baumannii (Red blood cell shaped)[31] (e) Vibrio cholera (coma shaped)[32] (f) Streptococcus pyogenes (oval shaped)[33] (g) Salmonella typhimurium (Rod shaped)[34] (h) Campylobacter fetus bacteria (rod shaped)[35]. (Scale bar (a) (d) and (f) 0.5 µm; (b)(c)(e)(g)(h) 2 µm).

Bacteria are the major cause of infectious diseases in humans. Infectious diseases caused by bacteria are the leading source for hospital visits across the world. An estimated 2 million deaths are reported every year due to tuberculosis caused by the mycobacterium tuberculosis alone whereas more than hundred million cases of pneumonia are reported annually [1], [2]. Bacteria exhibit a wide range of properties in terms of size, shape and surface biochemistry, all of which contribute to their virulence [3] (**Figure 1**). Elucidating the mechanism of bacterial invasion and clearance by the host immune system is essential for the treatment of such pathologies. In the human body, the mononuclear phagocytic system (MPS) that mainly comprises of macrophages, bone marrow progenitors and blood monocytes plays a central role in both innate and humoral immune defense against pathogens such as bacteria, cellular debris and foreign entities [4], [5]. Several studies have been performed to investigate the recognition and subsequent elimination of bacteria by macrophages [6], [7]. To this effect, a variety of receptors (toll-like receptors for innate immunity and Fc receptors for humoral immunity) have been identified on the macrophage surface that efficiently recognize different bacterial strains. However, the morphological features of bacteria, in particular the wide variety of sizes and shapes exhibited by bacteria, have not been given adequate consideration in recognition by macrophages.

Conversely, the geometry of drug delivery carriers has been recently recognized as an important design parameter [8] in addition to size [9], [10], bulk material [11], [12], surface chemistry [13], [14] and mechanical properties [15]. Importance of particle geometry in various biological functions such as phagocytic internalization [16], [17], transport in the vasculature [18] and blood circulation half life [19] has been illustrated. Findings of these studies suggest that the wide variety of bacterial shapes can also potentially play an important role in their recognition and clearance by the macrophages. Here, using polymeric particles of different geometries that represent the size and shape range of a variety of bacteria, we investigated the interactions of particles with the MPS. In particular, particles were coated with immunoglobulins (IgG) to simulate the opsonization process and their attachment to the macrophage surface was studied. Particles of different geometries indeed exhibited remarkably different attachment tendencies to the macrophage surface. Analysis of experimental data revealed the importance of surface features in recognition of target geometry by macrophages.

## Materials and Methods

### Particles

Polystyrene particles with green fluorescence of different sizes and shape were used to study attachment to macrophages. Spheres of three different sizes 0.5±0.012, 1±0.01 and 3±0.065 µm were purchased from Polysciences (Warrington, PA, USA) whereas rod and oblate ellipsoid-shaped particles were stretched from the spheres using the film-stretching technique described by Champion et al. [16]. In order to replicate opsonization *in vitro*, particles were passively coated with mouse IgG (Sigma Aldrich, St. Louis, MO, USA). In order to coat IgG on the surface, particles were incubated with 1 mg/mL solution of IgG for 2 hours at room temperature over a shaker plate. The particles were subsequently washed twice with PBS to remove any unbound IgG.

### Cells

J774 mouse monocyte macrophage cell line (ECACC products, Sigma Aldrich, St. Louis, MO, USA) was used to study attachment of particles. The cells were cultured with Dulbecco Eagle media (ATCC, Manassas, VA, USA) which was supplemented with 1% penicillin/streptavidin (Sigma Aldrich, St. Louis, MO, USA) and 10% fetal bovine serum (Invitrogen, Carlsbad, CA, USA). Cells were grown in standard culture conditions (37°C and 5% CO_2_).

### Attachment

The cells were seeded in 24 well plates at a concentration of 5×10^5^ per well and left overnight in the incubator. On the next day, the media was replaced with fresh media warmed to 37°C just prior to the experiment. The cells were then cooled to 4°C and particles with concentrations ranging from 10^6^ to 5×10^7^/well were added to the cells. The particles were allowed to attach at 4°C for 4 hours. In order to remove the unattached particles, the cells were washed 8 times with PBS at 4°C. The number of particles attached was quantified using fluorescence microscopy.

### Fluorescence Microscopy

The attachment of particles to the macrophages was quantified using an Axiovert 25 (Carl Zeiss Inc., Thornwood, NY) fluorescence microscope. The particles purchased from Polysciences were yellow-green fluorescent and were excited using a FITC filter. Brightfield and fluorescent images were taken using a CoolSNAPHQ CCD camera (Roper Scientific, Tucson, AZ) connected to Metamorph® software and overlayed. The images were analyzed using the Image J software to count the number of particles attached per cell.

### Scanning electron microscopy (SEM)

SEM was used to confirm attachment of particles on cells and observe the orientation of attachment of particles of different shapes. After four hours of particle incubation with the cells grown over glass coverslips at 4°C, the unbound particles were washed away and the cells were fixed with 2.5% glutaraldehyde. Cells were washed with increasing concentrations of ethanol (up to 100%), vacuum dried and coated with palladium (Hummer 6.2 Sputtering System, Anatech Ltd., Union City, CA). Cells were imaged with the Sirion 400 SEM (FEI Company, Hillsboro, OR) at an acceleration voltage of 2 kV.

## Results and Discussion

**Figure 2:**
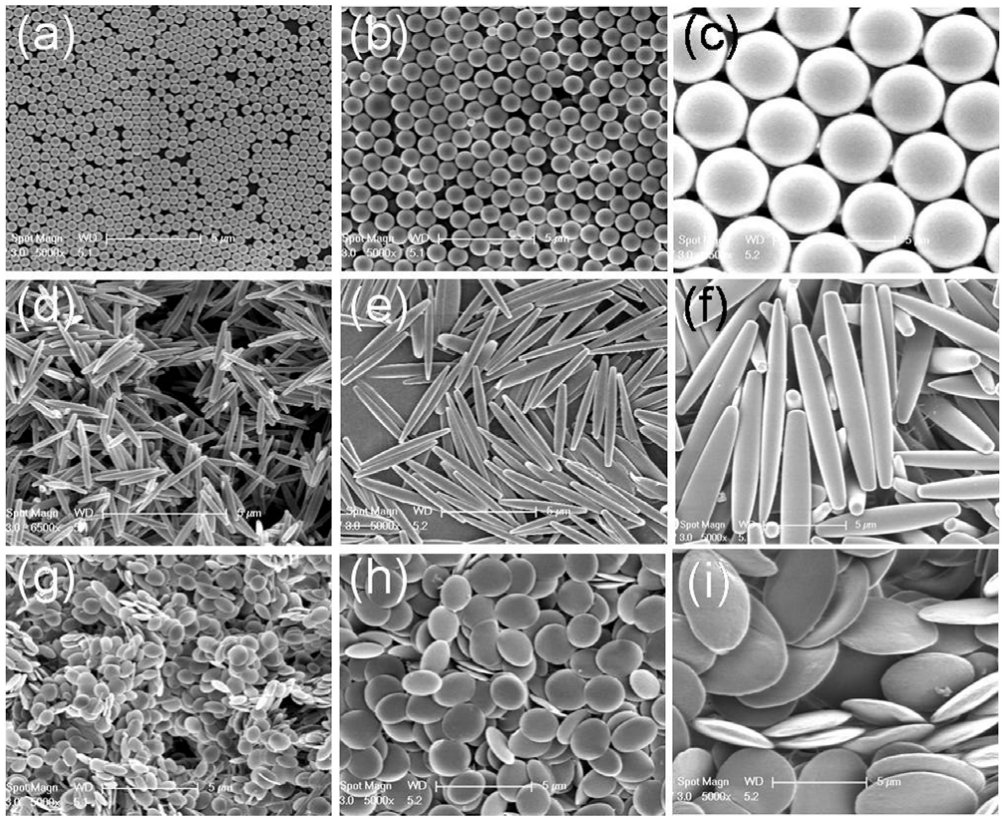
Scanning electron micrographs of the panel of particles of different geometries used for the attachment study. (a–c) Spheres of 0.5 µm, 1 µm and 3 µm respectively; (d–f) Rods stretched from 0.5 µm, 1 µm and 3 µm spheres respectively; (g–i) Oblate ellipsoids Rods stretched from 0.5 µm, 1 µm and 3 µm spheres respectively. The volume of the particles in a particular column is constant and corresponds to the volume of the sphere in that column. (Scale bar 5 µm for all images)

Using commercially available fluorescent spheres of three different sizes (0.5, 1 and 3 µm) and by stretching them to form rods and oblate ellipsoids [16], a panel of 9 different particle geometries with 3 different shapes and 3 different sizes was created (**Figure 2**). Since the volume of the particle remains constant during stretching, the three sets of particle shapes possessed a constant volume corresponding to 0.5, 1 and 3 µm spheres respectively. Since many bacteria are rod shaped [3], the rod shaped particles help elucidate the importance of bacterial geometry, in addition to drawing out the importance of particle elongation, in attachment to the macrophages. The oblate ellipsoidal shape is rarely found in bacteria but acts as a control non-spherical shape in this study. Particles were opsonized by coating them with mouse IgG and allowed to attach to mouse macrophage surface by incubation at 4°C, thereby avoiding internalization via phagocytosis. Internalization was avoided in this study since it has its own dependence on size and shape and has been previously reported [10], [16], [20]. The focus of this study was on recognition of size and shape as reflected in the attachment step.

**Figure 3:**
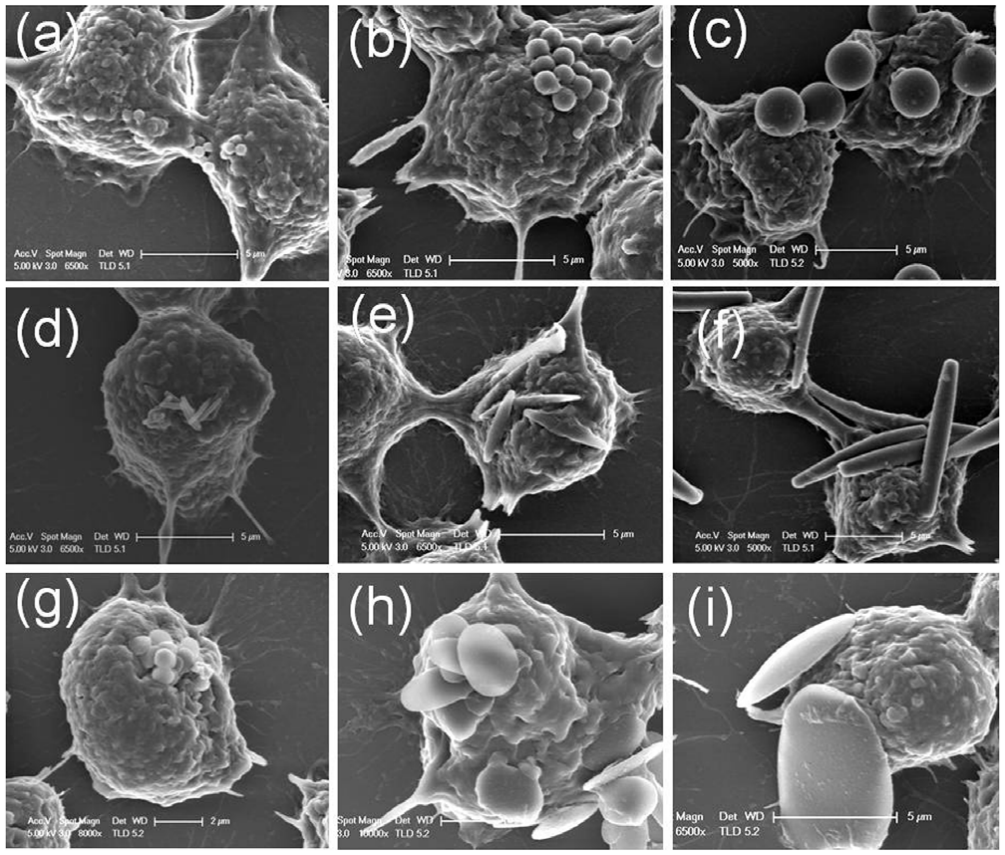
Scanning electron micrographs of attachment of particles of different geometries to the macrophages. (a–c) Spheres of 0.5 µm, 1 µm and 3 µm respectively attached to macrophages; (d–f) Rods stretched from 0.5 µm, 1 µm and 3 µm spheres respectively attached to macrophages; (g–i) Oblate ellipsoids stretched from 0.5 µm, 1 µm and 3 µm spheres respectively attached to macrophages. Scale bar (a, d, g, h: 2 µm; b, c, e, f, i: 5 µm).

**Figure 4:**
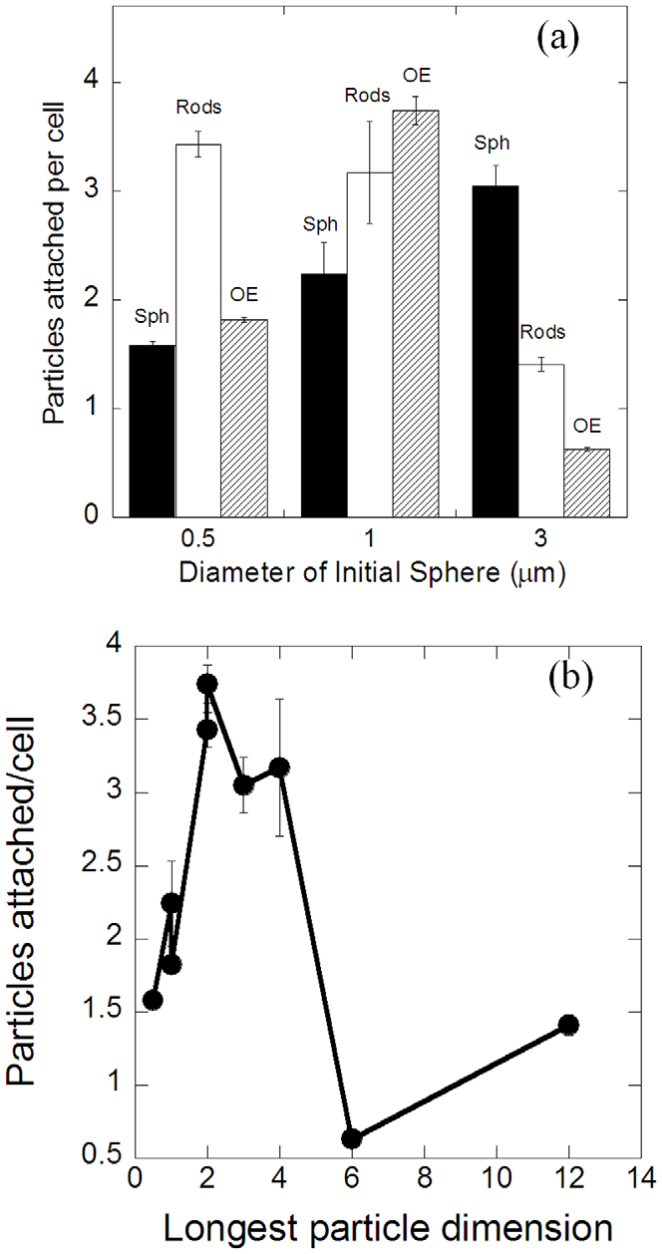
Attachment propensity of particles of different geometries. (a) Comparison of the attachment propensity of particles of different geometries quantified in terms of the number of particles attached per cell (number of cells counted  = 300), (b) Number of particles attached per cell plotted as a function of the largest particle dimension.

The attachment propensity depends strongly on the contact area between the particles and the macrophage surface. The SEM micrographs illustrate the orientation in which particles of different geometries attached to the macrophage surface (**Figure 3**). The attachment propensity was quantified in terms of number of particles attached per cell. Size and shape of the particle had a significant effect on the attachment propensity of particles (**Figure 4a**). For 0.5 µm, rod shaped particles exhibited significantly higher attachment compared to spheres and oblate ellipsoids. For 1 µm particles, oblate ellipsoids showed highest attachment tendency followed closely by rods and then by spheres. For 3 µm particles, an opposite trend was observed where spheres showed higher attachment compared to rods and oblate ellipsoids.

For particles fabricated from 0.5 µm spheres, the observations are consistent with the potential surface area of contact exhibited by these particles with rods having the largest surface area followed by oblate ellipsoids and spheres. For particles fabricated from 1 µm spheres, although rods possess higher surface area compared to oblate ellipsoids, the attachment tendency of oblate ellipsoids is higher, however the two are significantly higher than spheres which possess the least surface area. However, in case of 3 µm particles, the surface area of the particle is comparable to the macrophage surface area and attachment was therefore limited by the exposed surface area of the macrophage.

**Figure 5:**
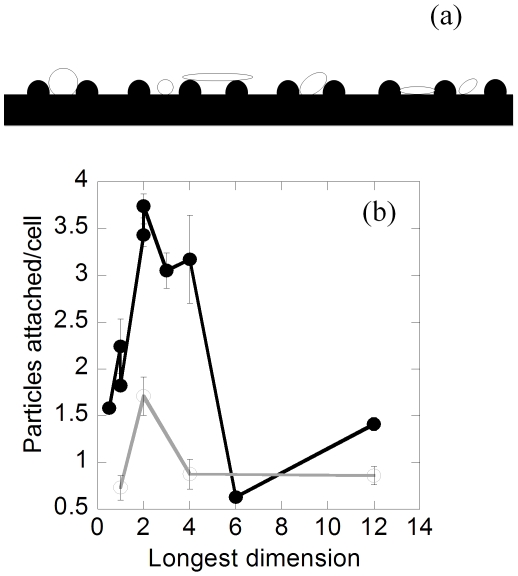
Role of membrane ruffles in particle attachment. (a) Schematic representation of membrane ruffles on the macrophage surface (black) that can potentially result in higher attachment of particles (white) of certain geometry, (b) Swollen macrophages exhibit a decrease in attachment propensity. Comparison of the number of particles attached per cell as a function of the longest particle dimension for normal (open circles) and swollen (solid circles) macrophages. There is a significant decrease in the peak for attachment propensity in case of swollen macrophages. (p<0.01)

The trends observed for particles of same shape but of different sizes cannot be explained solely in terms of the contact surface area. When plotted against the longest dimension (see Table S1), particle attachment exhibited a peak at around 2–3 µm for all sizes and shapes (**Figure 4b**). This observation is consistent with the observation reported by Champion et al. that the attachment of spherical particles of different diameters on the macrophage exhibits a peak at around 2–3 µm [10]. They further correlated the occurrence of the peak to the morphological features of the membrane ruffles present on the macrophage surface. Specifically, they proposed that the spacing and the size of membrane ruffles leads to optimal trapping of particles of intermediate sizes. Following the same argument, it can be postulated that the size, shape and orientation of the particle with respect to membrane ruffles can potentially affect the attachment tendency of the particle (**Figure 5a**). Particle and particle orientations that can fit well between the two membrane ruffles will exhibit stronger binding due to higher number of contact points with the macrophage. This is also consistent with the higher attachment of oblate ellipsoids compared to rods made from 1 µm spheres, although the surface area of rods is higher than that of oblate ellipsoids in this case.

In order to further test the validity of this hypothesis, the characteristic features on the mouse macrophage surface were eliminated by osmotic swelling of the cells. Osmotically swollen macrophages possess a smooth surface and the effect of membrane ruffles can thus be eliminated [10]. The attachment propensities of opsonized particles to swollen macrophages were significantly lower compared to their un-swollen counterparts (**Figure 5b**). The most drastic decrease in the attachment propensity was observed for the particles with the largest dimension in the 2–3 µm range which further testifies the hypothesis of the dependence of attachment on particle geometry and presence of membrane ruffles on the macrophages.

The importance of opsonization and particle geometry in internalization by macrophages has already been reported in literature [16], [21]. Internalization of particles has been shown to exhibit a strong dependence on the local geometry at the interface of particle and the cell [22]. The results of the studies presented here show that the attachment step also exhibits a peculiar dependence on particle size and shape.

**Figure 6:**
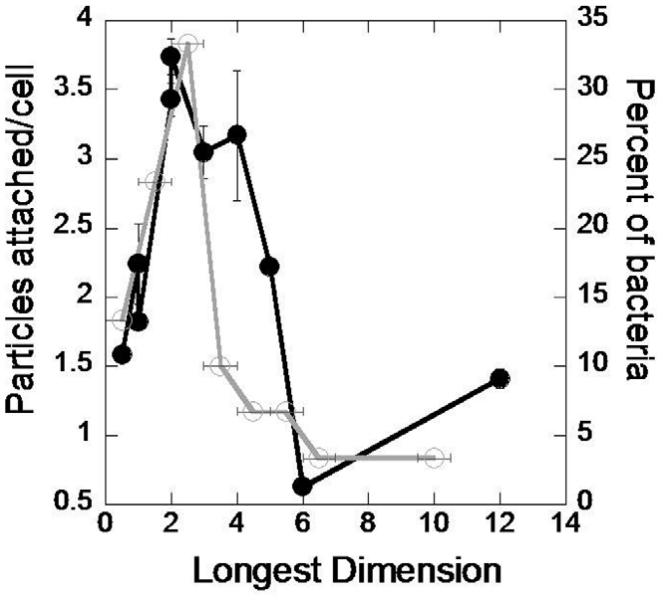
Comparison of the attachment propensities of particles to macrophages with the size distribution of bacteria. Black line represents number of particles attached per cell plotted as a function of the longest particle dimension whereas the grey line represents the percentage of bacterial population plotted as a function of the longest bacterial dimension.

In order to assess whether the role of geometry in particle attachment has any relevance to bacteria, we selected 30 common bacterial strains and evaluated their size and shape (Table S2). Most bacteria are rod shaped and vary in characteristic dimensions from a few nm to tens of µm. The longest dimension of the bacteria was determined and the distribution of the longest dimension of the bacterial strains was plotted. This distribution exhibited a peak around 2 µm, the length where particles exhibited highest attachment (**Figure 6**).

The attachment profiles of particles of different geometries and their striking resemblance to bacterial size distribution pose an important question: are these two related? Although a definitive answer may be difficult to obtain, the data point to the possibility that this might be the case. Understanding interactions between macrophages and their targets has been a topic of high interest. Towards that end, significant attention has already been paid to understanding receptor-mediated recognition of pathogens. The importance of opsonins in enhancing the rate of phagocytosis is well established [23]. CR1 and Fc receptor present on the macrophage surface bind to C3b and antibodies thereby facilitating phagocytosis of the target. Besides, macrophages express a wide range of pattern recognition receptors including CD14 and toll like receptors (TLRs) that enable detection of conserved microbial structures [24]. For example, TLR2 and TLR4 are implicated in the recognition of various bacterial cell wall components such as lipopolysaccharide, lipoproteins and glycolipids [25]. Moreover, the different TLRs co-ordinate macrophage activation to recognize a wide spectrum of stimuli [26]. Besides, the macrophages also execute an innate host surveillance mechanism that recognizes intracellular bacteria [27]. In contrast to the extensive research on receptor-mediated recognition of pathogens, the involvement of physical features such as target size and shape in the recognition process is not studied. Since macrophages first interact with the physical features of bacteria such as size and shape, it is reasonable that these properties play a critical role in their recognition. Contributions of physical and chemical features in bacterial recognition occur at different length scales. While the physical features such as size and shape span a much larger length scale (microns), receptor-ligand interactions occur at a smaller scale (nanometers). Hence, it is plausible that the two work in synergy to maximize pathogen recognition. It also appears likely that since the macrophages are body's innate means of countering pathogens, they might have evolved to recognize as many features of pathogens as possible. Given the strong patterns in size and shape of pathogens, development of capabilities to recognize pathogens based on physical features, in addition to surface biomolecules, perhaps represents an evolutionary step that the macrophages utilize in fighting bacterial infections.

In summary, using polymeric particles of different geometries, it is demonstrated that target geometry plays an important role in attachment to the macrophage surface, the first step in target elimination. Most bacteria are rod shaped and a large percentage have a longest dimension in the range of 2–3 µm. Particles with the longest dimension in the same range exhibited maximum attachment tendency to macrophages. Moreover, the surface ruffles on macrophage appear to play an important role in particle attachment. This leads to the hypothesis that macrophage surface features may be designed to recognize the shape of the bacteria thereby facilitating their clearance. This is a valuable hypothesis related to fundamental understanding of how the immune system is designed to counteract bacterial pathogenicity and should be further tested.

From biomedical applications point of view, with the advances in the design of carriers for drug delivery, it is important to study the interactions of carriers with the MPS. The significant differences in the attachment of opsonized particles of different geometries to macrophage surface illustrated by this study further underscore the growing importance of carrier geometry in various biological processes involved in the delivery of therapeutics.

## Supporting Information

Table S1 (0.03 MB DOC)

Table S2 (0.07 MB DOC)
