# Supplementary material for: Macrophages Recognize Size and Shape of Their Targets
Source: PLoS One. 2010 Apr 6;5(4):e10051. doi: 10.1371/journal.pone.0010051 (PMC2850372; doi:10.1371/journal.pone.0010051)
Supplement: Table S1 — (0.03 MB DOC) [file pone.0010051.s001.doc]

**Table SI**: Largest particle dimension used in this study.

| **Particle** | **Average largest dimension (μm)** |
| --- | --- |
| 0.5 μm Spheres | 0.5 |
| Rods from 0.5 μm spheres | 2 |
| Oblate Ellipsoids from 0.5 μm spheres | 1 |
| 1 μm Spheres | 1 |
| Rods from 1 μm spheres | 4 |
| Oblate Ellipsoids from 1 μm spheres | 2 |
| 3 μm Spheres | 3 |
| Rods from 3 μm spheres | 12 |
| Oblate Ellipsoids from 3 μm spheres | 6 |
