# Supplementary material for: Macrophages Recognize Size and Shape of Their Targets
Source: PLoS One. 2010 Apr 6;5(4):e10051. doi: 10.1371/journal.pone.0010051 (PMC2850372; doi:10.1371/journal.pone.0010051)
Supplement: Table S2 — (0.07 MB DOC) [file pone.0010051.s002.doc]

**Table SII: List of common bacteria** [1,2,3,4,5,6,7,8,9,10,11,12,13,14,15,16,17]

| **No** | **Bacteria Name** | **Dimensions (μm)** | **Longest dimension averaged (μm)** |
| --- | --- | --- | --- |
| 1 | Acinetobacter baumannii | 0.8 | 0.8 |
| 2 | Alcaligenes Viscolactis | 0.6-1.0x0.8-2.6 | 1.5 |
| 3 | Acetobacter Melanogenus | 0.4-0.8x1.0-2.0 | 1.5 |
| 4 | Bacillus cereus | 1x3-4 | 3.5 |
| 5 | Bacillus subtilis | 0.7-0.8x2.0-3.0 | 2.5 |
| 6 | Bacillus Stearothermophilus | 0.6-1.0x2.0-5.0 | 3.5 |
| 7 | Bacillus anthracis | 1.0-1.3x3.0-10.0 | 6.5 |
| 8 | Borrelia burgdorferi | 0.5 x 10 | 10 |
| 9 | Bordetella pertussis | 0.4 x 0.8 | 0.8 |
| 10 | Campylobacter fetus bacteria | 0.4x1-3 | 2 |
| 11 | Chlamydia pneumoniae | 0.5-1 | 0.75 |
| 12 | Clostridium tetani | 0.5-0.8x3-8 | 5.5 |
| 13 | Clostridium Perinngens | 1.0-1.5x4.0-8.0 | 6 |
| 14 | Clostridium difficile | 2x5 | 5 |
| 15 | Erwina aroideae | 0.5x1-3 | 2 |
| 16 | Escherichia coli | 0.5 x 1-3 | 2.5 |
| 17 | genus mycoplasma | 0.1-0.2 | 0.2 |
| 18 | Haemophilus influenzae | 0.2-0.3x0.5-2 | 1.5 |
| 19 | Helicobacter pylori | 0.5 x 2-3 | 2.5 |
| 20 | Lactobacillus Delbrueckil | 0.5-0.8x2-7 | 4.5 |
| 21 | Listeria monocytogenes | 0.5 x 2-3 | 2.5 |
| 22 | Klebsielia pneumoniae | 0.4-0.5x5 | 3 |
| 23 | Mycobacterium leprae | 0.5 x 1-3 | 2 |
| 24 | Mycobacterium tuberculosis | 0.5 x 2 | 2 |
| 25 | Mycoplasma pneumoniae | 0.25 x 1 | 1 |
| 26 | Neisseria gonorrhoeae | 1 | 1 |
| 27 | Salmonella typhi /paratyphi | 0.5 x 2 | 2 |
| 28 | Staphylococcus aureus | 1 to 2 | 1.5 |
| 29 | Streptococcus pyogenes | 0.5x1.2 | 1.2 |
| 30 | Vibrio cholerae | 0.5 x 2 | 2 |

**References**
